# A 30-Min Exposure on Permethrin and Deltamethrin Modifies Ion Transport Pathways in the Skin

**DOI:** 10.3390/biom14121491

**Published:** 2024-11-22

**Authors:** Karolina Szewczyk-Golec, Katarzyna Mądra-Gackowska, Łukasz Szeleszczuk, Jan Szczegielniak, Iga Hołyńska-Iwan

**Affiliations:** 1 Department of Medical Biology and Biochemistry, Faculty of Medicine, Ludwik Rydygier Collegium Medicum in Bydgoszcz, Nicolaus Copernicus University in Torun, 85-067 Bydgoszcz, Poland; karosz@cm.umk.pl; 2 Department of Geriatrics, Faculty of Health Sciences, Ludwik Rydygier Collegium Medicum in Bydgoszcz, Nicolaus Copernicus University in Torun, 85-067 Bydgoszcz, Poland; katarzyna.madra@cm.umk.pl; 3 Department of Organic and Physical Chemistry, Faculty of Pharmacy, Medical University of Warsaw, 02-091 Warsaw, Poland; lukasz.szeleszczuk@wum.edu.pl; 4 Physiotherapy Department, Faculty of Physical Education and Physiotherapy, Opole University of Technology, 45-758 Opole, Poland; j.szczegielniak@po.edu.pl; 5 Ministry of Internal Affairs and Administration’s Specialist Hospital of St. John Paul II, 48-340 Glucholazy, Poland; 6 Laboratory of Electrophysiology of Epithelial Tissue and Skin, Department of Pathobiochemistry and Clinical Chemistry, Faculty of Pharmacy, Ludwik Rydygier Collegium Medicum in Bydgoszcz, Nicolaus Copernicus University in Torun, M. Skłodowskiej-Curie 9, 85-094 Bydgoszcz, Poland

**Keywords:** chloride, ion transport, pyrethroid, resistance, skin, sodium

## Abstract

Pyrethroids are pesticides used in agriculture, the textile industry, wood processing, and human and animal medicine. Pyrethroids inhibit voltage-sensitive sodium channels (VSSCs) in insects and mammals. It results in the premature opening and/or delayed closing of the channels, causing a prolonged influx of Na^+^ ions into the cell. Insects absorb pyrethroids throughout the entire body surface, while poisoning in humans most often occurs by inhalation and through the skin. In this study, 52 fragments of human skin taken from the eyelid fold were examined. A modified Ussing chamber was used to measure the active ion transport in epithelial tissue and quantify the tissue viability and integrity. Both permethrin and deltamethrin solutions induced changes in the transport of ions, mainly sodium, but by different mechanisms. Permethrin affected the transepithelial transport of sodium ions in a long-term mechanism, while deltamethrin affected the ability to respond to stimuli in an immediate mechanism. Contact with deltamethrin may cause a delay/slowness of sensation, inflammation, hypersensitivity, and/or allergy. The action of permethrin takes place in the intercellular spaces and is associated with the possibility of faster decomposition/metabolism, while deltamethrin interacts with receptors, channels, and the cell membrane, which translates into slower decomposition and longer action in the tissue.

## 1. Introduction

Pyrethroids, derivatives of chrysanthemum acids, are pesticides commonly used in the agriculture, textile, and wood industries, as well as in disease treatment and prevention in humans and animals [1,2,3,4,5,6]. As a result of the World Health Organization (WHO)’s recommendations for the prevention and control of insect-borne diseases, the use of pyrethroids and the number of people exposed to these compounds are increasing [7,8,9]. Pyrethroids are considered generally safe for vertebrates, including humans [10,11,12,13]. In mammals, pyrethroids are relatively quickly broken down into harmless metabolites in the liver [14]. Pyrethroids are lipid-soluble, which allows them to enter the body via transepidermal and then transcellular transport [13,14,15]. Insects absorb pyrethroids with their entire body surfaces [13]. The lower toxicity of pyrethroids to mammals is due to their larger body surface areas and different body temperatures, which make pyrethroids approximately 2250 times less toxic to mammals than to insects [10,12]. Poisoning in humans occurs relatively rarely, most often through inhalation and through the skin [1,3,15].

**Figure 1:**
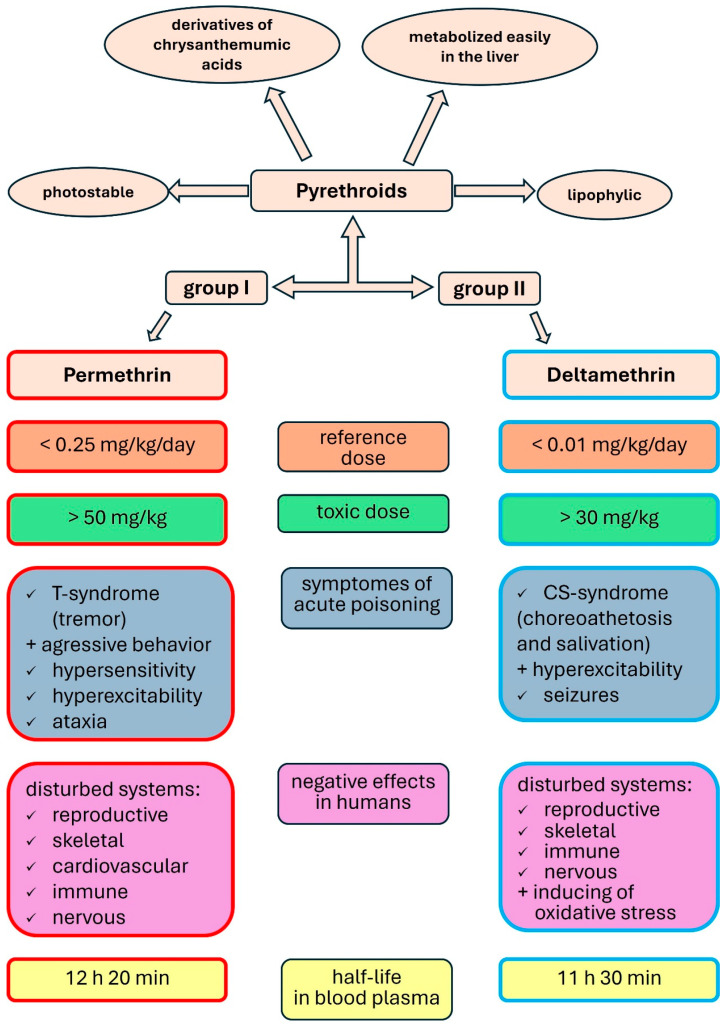
Characteristics of permethrin and deltamethrin.

Pyrethroids have been divided into two groups based on the mechanisms of their toxic actions [11,16,17]. Permethrin belongs to group I pyrethroids. Permethrin poisoning in humans causes parasthesia, skin irritation and itching, throat and abdominal pain, nausea and vomiting, headaches, mucous membrane irritation, breathing problems, and dizziness [11,16,17]. Deltamethrin is classified as a pyrethroid of group II. Deltamethrin poisoning causes vomiting and diarrhea, muscle tremors, heart rhythm disturbances with tachycardia, cough, runny nose, pulmonary edema, blurred vision, somnolence, and convulsions [11,16,17]. When in contact with the skin, deltamethrin may cause an allergic reaction characterized by rash, itching, burning, redness, and irritation of the mucous membranes [1,6,18]. The characteristics of permethrin and deltamethrin are presented in Figure 1.

The mechanism of pyrethroid action includes the inhibition of voltage-sensitive sodium channels (VSSCs) in insects [13] and mammals [19]. VSSC inhibition causes the premature opening and/or delayed closure of the channels, resulting in a prolonged inflow of Na^+^ ions into the cell [11]. Therefore, a higher potential is needed to close the sodium channels during hyperpolarization than during physiological conditions [11], which forces the cells to generate a higher frequency of depolarization, increasing their sensitivity to stimuli [11,13,18]. In addition to VSSCs, deltamethrin also affects chloride and calcium channels [18,20].

Analysis of the changes in the electrophysiological parameters in skin specimens with preserved layering, reactivity, and sensitivity to stimuli makes it possible to observe physiological reactions and those reactions changed by xenobiotics in the pathways of ion and water transport [21,22,23,24]. Epithelial sodium channels (ENaCs) involved in the transport of sodium ions and water, as well as the cystic fibrosis transmembrane conductance regulator (CFTR) channels and other chloride channels (ClCs), are active in keratinocytes [23,24,25,26,27]. Changes in the functioning of sodium and/or chloride channels may be associated with the development of hypersensitivity and/or allergies [23,28], problems with regeneration and healing [29], increased pain perception [30,31], and pigmentation disorders [32].

The number of incidents of both immediate and prolonged contact of repellants and insecticides with the human skin surface is constantly increasing. The pathomechanism of the action of group I and II pyrethroids on the skin has not been thoroughly described. In particular, there are no data on the effects of permethrin or deltamethrin on ion transport in the human skin. Therefore, the aim of the present study was to determine the impact of a 30-min incubation of human skin specimens in solutions of permethrin and deltamethrin, with low concentrations of 0.01 mM, on the transport of ions in the skin and its vitality, compactness, and ability to respond to stimuli.

## 2. Materials and Methods

The experiments were carried out in a modified Ussing chamber on specimens of human skin. This technique was developed to measure changes in ion transport in the epithelial tissue and to quantify tissue reactivity, viability, and integrity.

### 2.1. Patients

Human skin specimens (*n* = 52) were collected from the eyelid folds of patients of the Department of Plastic, Reconstructive, and Aesthetic Surgery of the University Hospital No. 1., Dr. A. Jurasz, in Bydgoszcz, Poland, with the consent of the Bioethics Committee of the Nicolaus Copernicus University in Toruń, functioning at Collegium Medicum in Bydgoszcz, Poland (no. KB 218/2015, of 12 March 2015). The patients, aged 50–65 years, including 12 women and 6 men, were operated on under aseptic conditions, observing the principles of art, due to non-inflammatory eyelid disorders (a diagnosis code of 9A06.Y according to the 11th version of the International Statistical Classification of Diseases and Related Health Problems [33] or H02 according to 10th version of the International Statistical Classification of Diseases and Related Health Problems [34]). They were assessed by the attending physician as being in good health, did not suffer from any chronic or acute diseases, and did not confirm any previous contact with pesticides. The characteristics of the patients are presented in Table 1. A specimen of the skin from each eyelid was collected from every patient. The prepared skin specimens were alive, with preserved layering and nerve endings, and did not contain muscle or adipose tissue.

### 2.2. Reagents

Reagents and solutions used in the experiment:

- RS—Ringer’s solution: Cl^-^ 160.8 mM; Na^+^ 147.2 mM; K^+^ 4.0 mM; Mg^2+^ 2.6 mM; Ca^2+^ 2.2 mM; 4-(2-Hydroxyethyl)piperazine-1-ethanesulfonic acid (Sigma–Aldrich, Burlington, NJ, USA), pH = 7.4, iso-osmotic basic solution. Used for incubation and mechanical stimulation.
- Perm—Permethrin (3-phenoxyphenyl)-methyl] 3-(2,2-dichloroethenyl)cyclopropane-1-carboxylate; mixture of cis- and trans-isomers, with a ratio of 1:3, 391.3 g/mol (Sigma–Aldrich, Burlington, NJ, USA), dissolved and diluted (0.01 mM, 3.9 mg/mL) in RS.
- Delta—Deltamethrin [(*S*)-Cyano-(3-phenoxyphenyl)-methyl]-(1*R*,3*R*)-3-(2,2-dibromoethenyl)-2,2-dimethyl-cyclopropane-1-carboxylate; 505.2 g/mol (Sigma–Adrich, Burlington, NJ, USA), dissolved and diluted (0.01 mM; 5.1 mg/mL) in RS.

### 2.3. Experimental Procedure

After collection, the tissue samples were kept in RS for up to 3 h; then, after being transported to the Laboratory of Electrophysiology of Epithelial and Skin Tissue, they were divided randomly, cut into fragments with an area of approximately 0.3 cm^2^, and incubated in the RS, Perm, or Delta solution. Incubation lasted 30 min and took place in darkness at a temperature of 25 °C and a constant humidity of 55%. The incubation time, temperature, and humidity were selected to mimic conditions in which humans may come into contact with insecticides. The time of 30 min was sufficient for the penetration of pyrethroids through the skin [15].

**Figure 2:**
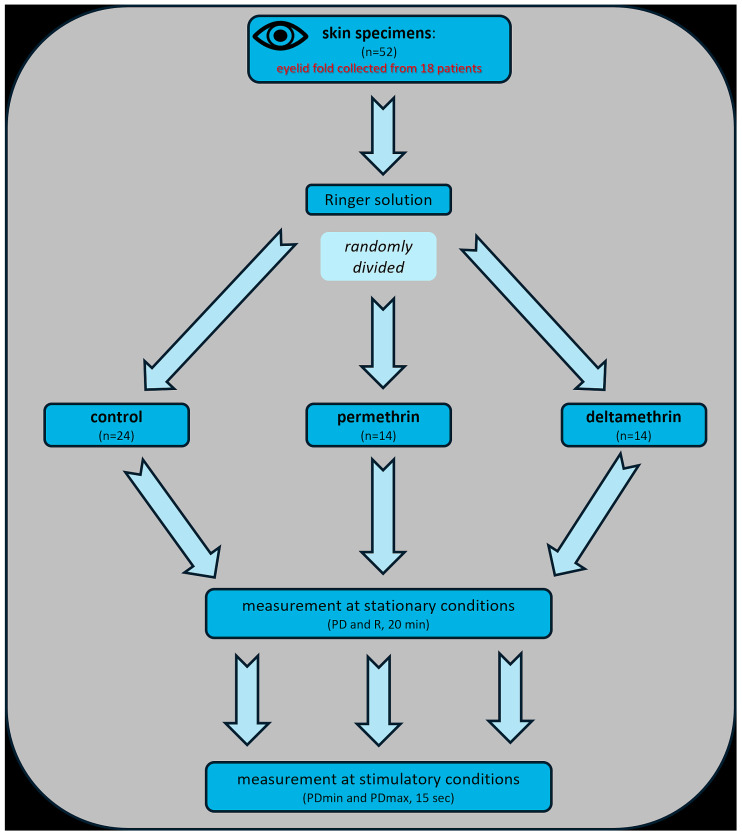
Study design. Abbreviations: PD—transepithelial electric potential measured at stationary conditions, R—transepithelial resistance, PDmin—minimal transepithelial electric potential measured during a 15-s stimulation, and PDmax—maximal transepithelial electric potential measured during a 15-s stimulation.

**Figure 3:**
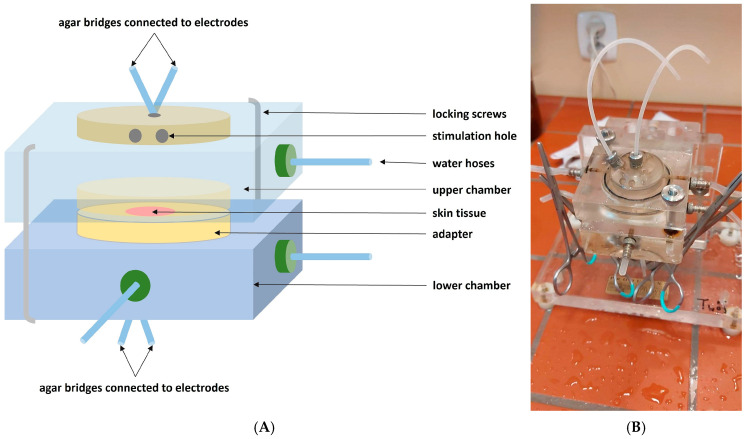
Modified Ussing chamber: (**A**) a scheme and (**B**) an original photo.

Each specimen was exposed to 1 mL of a solution of one pyrethroid. After incubation, the specimens were mounted in the modified Ussing chamber to measure the electrophysiological parameters (Figure 2). The connection of the chamber with the preamplifier, amplifier, and measuring equipment was provided by pairs of Ag/AgCl electrodes and agar bridges. The modification of the chamber included the arrangement of a tissue sample in a horizontal position, with the stratum corneum upward and toward a stimulation nozzle (the scheme presented in Figure 3). For the duration of a stimulation, a nozzle connected to a peristaltic pump was attached to the chamber, from which a fluid flowed out of the peristaltic pump at a volume of 0.06 mL/s (1 mL/15 s) and a temperature of 25 °C from a distance of 3–5 mm from the tissue surface, imitating drops falling on the tested skin’s surface. Mechanical–chemical stimulations with Perm or Delta and mechanical stimulations with RS were performed. The chambers were filled with Perm or Delta solutions, respectively. The area of the examined tissue samples was 0.25 cm^2^. The measurement of the electrophysiological parameters lasted 15 min.

The experiments consisted of measuring the following parameters:

1. Stationary conditions:
  - R—transepithelial resistance measured after applying a stimulus current of ±10 μA to the tissue sample; after measuring the voltage, the resistance was calculated according to Ohm’s law (Ω/cm^2^).
  PD—transepithelial electric potential measured continuously under stationary conditions (mV).
2. Stimulation conditions:
  - PDmin—minimal transepithelial electric potential measured during 15 s of mechanical (RS) and/or mechanical–chemical (Perm or Delta) stimulations (mV).
  PDmax—maximal transepithelial electric potential measured during 15 s of mechanical (RS) and/or mechanical–chemical (Perm or Delta) stimulations (mV).

Data were recorded using the EVC4000 experimental protocol (WPI, Worcester, MA, USA). It was connected to the data acquisition system and transferred to the AcqKnowledge 3.8.1 computer software (Biopac Systems, Inc., Goleta, CA, USA). The data of all measurements performed for every study group were calculated and analyzed. Statistical analysis was performed in Statistica 11.00 (StatSoft, Polska, Kraków, Polska). In order to determine the data distribution, the Kolmogorov–Smirnov test with Lilliefors corrections was used. The Mann–Whitney test and the Wilcoxon test were used with a *p*-value < 0.05.

## 3. Results

The skin specimens treated with Delta showed significantly lower R values than those recorded for the fragments treated with Perm (*p* < 0.05 in the Mann–Whitney test, Figures S1 and S2). R did not change significantly for any of the pyrethroids used during the experiment (Wilcoxon test, Table 2). No statistically significant differences were observed when comparing the R of the control tissue samples with those treated with pyrethroids (Table 2, Mann–Whitney test).

The control tissue samples and those exposed to the Perm solution showed no significant changes in continuous ion transport, measured as PD, which was −0.15 mV (median) during the experiment. The PD measured for Delta was significantly lower both at the beginning and the end of the experiment, but the PD significantly increased in the electropositive direction during the study (Table 3). No statistically significant differences were observed in the PD of the skin specimens treated with RS and incubated in pyrethroids (Table 3, Mann–Whitney test, Figures S3 and S4).

**Figure 4:**
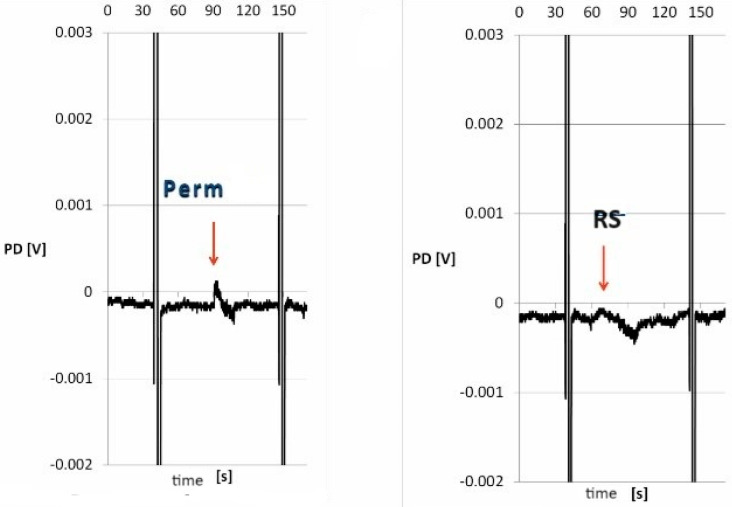
Exemplary diagram of changes in the transepithelial electrical potential during a 15-s mechanical–chemical (Perm) and a mechanical (RS) stimulation for a single skin fragment exposed to the permethrin solution for 30 min. Abbreviations: RS—iso-osmotic Ringer’s solution, Perm—permethrin solution (0.01 mM) in RS, PD—transepithelial electric potential measured in stationary conditions, ↓—start of the 15-s stimulation, and vertical lines—resistance.

**Figure 5:**
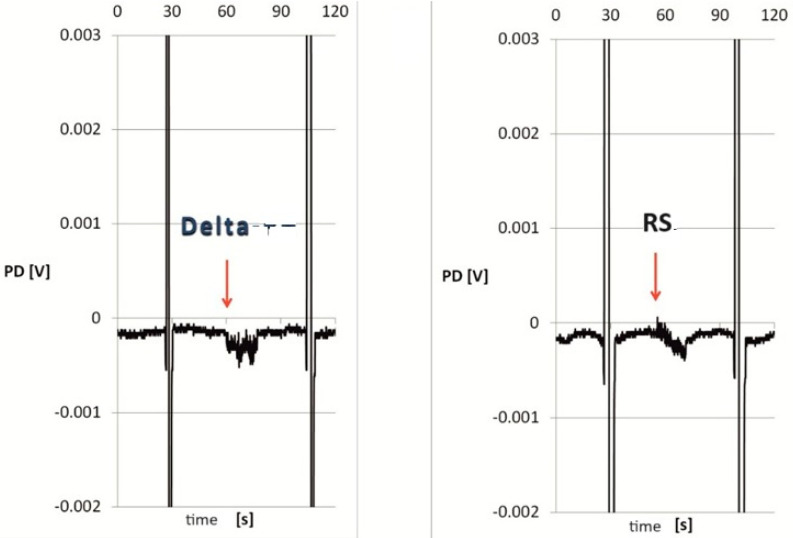
Exemplary diagram of changes in the transepithelial electrical potential during a 15-s mechanical–chemical (Delta) and a mechanical (RS) stimulation for a single skin fragment exposed to the deltamethrin solution for 30 min. Abbreviations: RS—iso-osmotic Ringer’s solution, Delta—deltamethrin solution (0.01 mM) in RS, PD—transepithelial electric potential measured in stationary conditions, ↓—start of the 15-s stimulation, and vertical lines—resistance.

The skin specimens treated with both Perm and Delta remained viable and reactive. The use of the mechanical–chemical stimulation (Perm, Delta) and the mechanical stimulation (RS) induced repeatable changes in ion transport, which were different from those recorded in stationary conditions, i.e., without any stimulation (Wilcoxon test, Table 4). The PDmin and PDmax for both the Delta and RS stimulations showed values higher than those for Perm, and the differences among the individual stimulations were insignificant. However, the fragments treated with Delta were significantly more reactive as compared to the fragments incubated in Perm (Mann–Whitney test, Table 4). Moreover, the Delta stimulation did not generate electropositive PDmax, unlike the Perm or RS stimulation (regardless of the pyrethroid used for the incubation). Interestingly, the responses to Perm were significantly lower as compared to Delta (Figure 4 and Figure 5).

## 4. Discussion

The modified Ussing chamber was used to examine tissue reactions to chemical and mechanical stimuli [18,22]. Changes in the transepithelial electrical potential reflected the ability of the tissue to adapt to a changing environment in terms of the transport of ions and water and the ability to respond to external stimuli [18,21,22]. The vitality, compactness, and water permeability of the full thickness of the skin fragments installed in the chamber were also tested. During the experiment, tissue samples were not exposed to damage and were washed by tested solutions, which imitated the conditions found in the environment [18,22]. The pathomechanism of the action of xenobiotics in contact with the skin most often begins with modifications in the transport of ions in the skin cells [25,35]. The effect of permethrin from pyrethroid group I and deltamethrin from group II, i.e., two pyrethroids with different chemical structures and different poisoning effects, on human skin has not been described so far.

The measurement of PD in stationary, unstimulated conditions reflects the constantly occurring ion transport, i.e., the operation of the sodium–potassium pump and the opening/closing of channels necessary to maintain the difference in the ion concentrations and the appropriate amount of water in cells and the spaces between them [22,23,24,26,27]. The penetration and effect of pyrethroids on the skins of mammals [15], including humans [3], has been described. Therefore, it should be recognized that in the present study, the Perm and Delta solutions affected not only the superficial layers of the skin, but, due to the penetration of pyrethroids into the deeper layers, this effect also covered the entire skin fragment subjected to the experiment. The Perm and Delta solutions were found to modify the transepithelial ion transport after 30 min of contact with the skin. The Perm solution with a concentration of 0.01 mM did not change the action of the sodium–potassium pump and the constant ion transport paths that occurred in individual skin layers; the measured PD values were constant throughout the whole experiment. This is consistent with the literature data [11,13], which demonstrated an effect on sodium channels, not the sodium–potassium pump. The rinsing effect of RS did not change the interaction of Perm with the examined tissue samples. The PD of the fragments incubated in permethrin was significantly higher as compared to that measured for the fragments incubated in Delta. The increase in the tissue surface’s electronegativity after the Delta treatment may have been caused by the intensification of the constantly occurring transport of ions related to the action of the sodium–potassium pump, as well as by influencing the secretion of chloride ions from the cell through an interaction with chloride channels [11,20]. It might have caused the prolonged opening of chloride channels, which interacts with the inflow of ions, especially sodium, into the cells [11,13]. Rinsing with RS resulted in the equalization of PD; therefore, the effect of Delta was reversible (Table 3). It could be concluded that after contact with pyrethroids, washing the body, i.e., rinsing with an aqueous solution, may be important for flushing these compounds from the skin and thus reducing their negative effects on the skin [3,6,35].

The reaction of the skin specimens stimulated for 15 s with permethrin solution did not change the ability of the tissue to respond to stimuli; after the RS administration, no statistically significant changes were observed in the value of the potential measured during the stimulation (PDmax, PDmin). However, the incubation in Delta changed the transport of sodium ions in response to the applied mechanical stimulus. Deltamethrin intensified the ion transport during the reaction to the mechanical stimulus; sodium ions probably flowed into the cells in larger amounts and/or chloride ions flowed out of the cells. PDmax increased significantly, which is related to the effect of deltamethrin on sodium channels and is analogously related to the effect of nerve endings in mammals and insects [11,13]. Chloride ions were probably retained or secreted on the surface of keratinocytes, which also confirms the effect of deltamethrin on chloride channels [13,16]. Changes in the ability to respond to stimuli and changes in the influx of sodium into cells are associated with the impaired intracellular transport of water [23] and the formation of micro-spaces. Micro-spaces may attract immunocompetent cells and cause an inflammatory reaction that results in the development of hypersensitivity and/or allergy [24]. Additionally, in their review, Soderlund et al. [13] described allergic reactions associated with other pyrethroids, including cypermethrin, bifenthrin, tefluthrin, and esfenthroin.

Both the permethrin and deltamethrin solutions induced changes in the transport of ions, mainly sodium ones, but the mechanism of the action of the tested pyrethroids differed. Permethrin affects the transepithelial transport of sodium ions in a long-term mechanism, while deltamethrin affects the ability to respond to stimuli primarily during a stimulation; i.e., it changes the ability to respond to stimuli during immediate action. Contact with deltamethrin may therefore result in a delay and/or slowness in sensation, as well as the development of inflammation, hypersensitivity, and/or allergy [1,2,10]. A similar effect of a 0.1 mM deltamethrin solution was described in short-term contact with rabbit skin [18]. The action of permethrin takes place in the intercellular spaces and is associated with the possibility of faster permethrin decomposition/metabolism, while deltamethrin interacts with receptors, channels, and the cell membrane, which translates into the slower decomposition of deltamethrin and its longer action within the tissue.

The resistance of mammal skin is characterized by high values [18,21,22,36,37,38,39]. In the present study, after the incubation in the Perm solution, the skin showed statistically significant higher resistance values as compared to the fragments incubated in the Delta solution for the measurements taken both at the beginning and at the end of the experiment. None of the compounds caused damage to the intercellular connections in the skin, microdamage, or deformations [22,24,38,39]. Therefore, pyrethroids did not affect the viability and integrity of the tested specimens [21,37,38]. The increased resistance after contact with permethrin may have resulted from the effect on the intracellular transport of sodium ions and water, causing an increased cell adhesion. The incubation for 30 min in the Perm solution could have resulted in lower ion permeability through the full-thickness skin fragments, which may be related to the dehydrating effect of permethrin on the intercellular spaces. In turn, the lower R values measured for the tissue samples incubated in Delta may indicate the increased permeability of the cell membranes to ions and water, the loosening of the spaces between cells, and the increased flow of ions between the skin layers (Table 2). In studies describing the short-term effect of Delta solutions on rabbit skin, no changes in resistance have been observed [18].

Humans and other species may be exposed to pyrethroids at the concentrations described in this experiment on a daily basis. Spraying crops or standing near them results in skin contact with deltamethrin [3,6,17], whereas the use of insect nets, insecticides, insect repellents, or lice shampoos involves the direct application of permethrin to the skin [12,39,40]. The LD50 of pyrethroids for permethrin in animals is 0.5 to 2 g/kg body mass and is 0.2 to 0.5 mg/kg body mass for deltamethrin [7]. In shampoos, permethrin doses of 500 mg/mL of the product are used; in insecticides, the dose can be up to 400 mg/mL of the product [40]. In the case of deltamethrin in preparations, 100 g/l of the product is used, which translates into the recommended 0.2 g/ha for spraying [6].

A limitation of this study is its evaluation of the effect of pyrethroids on the skin of the eyelids. The skin of the eyelids is thin as compared to the skin on the cheeks or abdomen and does not contain hair follicles, unlike the scalp. Skin collected from different sites, such as the hands, feet, or thighs, may respond differently to xenobiotics due to the number of cell layers, the presence of glands, or nerve endings [41]. However, the applied research model seems realistic due to facial exposure, direct contact and the involuntary touching of the face, including the eye area, which may result in the more frequent transmission of pyrethroids or other xenobiotics.

## 5. Conclusions

The growing need for protection against insects generates a high frequency of contact with pesticides, including pyrethroids, which, despite causing undesirable symptoms in humans, are considered one of the safer protective agents. In the present study, it has been proven that permethrin and deltamethrin, after 30 min of incubation, even at a low concentration of 0.01 mM, can cause changes in ion transport in human skin. Deltamethrin in humans, as in insects, appears to affect sodium and chloride channels, but it also seems to increase the permeability of ions and water through individual layers of the human skin. The effect of the permethrin solution on ion transport in human skin was less expressed as compared to that of deltamethrin, and it was concentrated in intracellular spaces. Due to the fact that we cannot avoid contact with pyrethroids, avoiding direct contact of these pesticides on the naked skin and mucous membranes and practicing thorough washing seems to be the right approach.

## Figures and Tables

**Table 1:** Patient characterisctics.

Number of patients	18
Sex (female/male)	12/6
Age (years)	50–65 (56 median)
Body mass index (kg/m^2^)	<30
Diet	Standard *
Well-condition	Standard *
Stress	Standard *
Smoking	No
Acute/Chronic diseases	No
Medicines for internal/external use	No
Physical examinations	No deviations
Contact with pesticide (e.g., home, work, garden, cultivated fields)	No

* Standard—means not exceeding the recommendations of the World Health Organization.

**Table 2:** Values of transepithelial resistance (R) of the skin specimens exposed to the permethrin (Perm, 0.01 mM) or deltamethrin (Delta, 0.01 mM) solution for 30 min.

	Parameter	R (Ω/cm^2^)	Wilcoxon Test (*p*) R Initial vs. R Final
Control (*n* = 24)	R initial	1567	0.3491
	R final	1120	
Perm (*n* = 14)	R initial	1691	0.7299
	R final	1800	
Delta (*n* = 14)	R initial	788	0.2209
	R final	740	
Mann–Whitney test (*p*) Control vs. Perm	R initial vs. R initial		0.2953
	R final vs. R final		0.2155
Mann–Whitney test (*p*) Control vs. Delta	R initial vs. R initial		0.7901
	R final vs. R final		0.8451
Mann–Whitney test (*p*) Perm vs. Delta	R initial vs. R initial		0.0208
	R final vs. R final		0.0192

*p* < 0.05 is considered statistically significant (in red).

**Table 3:** Values of transepithelial electric potential (PD), measured in stationary conditions, of the skin specimens exposed to the permethrin (Perm, 0.01 mM) or deltamethrin (Delta, 0.01 mM) solution for 30 min.

	Parameter	PD (mV)	Wilcoxon Test (*p*) PD Initial vs. PD Final
Control (*n* = 24)	PD initial	−0.21	0.2446
	PD final	−0.22	
Perm (*n* = 14)	PD initial	−0.15	0.0634
	PD final	−0.15	
Delta (*n* = 14)	PD initial	−0.33	0.0306
	PD final	−0.27	
Mann–Whitney test (*p*) Control vs. Perm	PD initial vs. PD initial		0.2771
	PD final vs. PD final		0.8995
Mann–Whitney test (*p*) Control vs. Delta	PD initial vs. PD initial		0.1359
	PD final vs. PD final		0.5047
Mann–Whitney test (*p*) Perm vs. Delta	PD initial vs. PD initial		0.0288
	PD final vs. PD final		0.0389

*p* < 0.05 is considered statistically significant (in red).

**Table 4:** Values of the minimal (PDmin) and maximal (PDmax) transepithelial electrical potential measured during a 15-sec stimulation of the skin fragments exposed to the permethrin (Perm, 0.01 mM) or deltamethrin (Delta, 0.01 mM) solution for 30 min.

	Stimulation Conditions			Wilcoxon Test (*p*)		
	PDmin (mV)	PDmax (mV)	PDmax–PDmin (mV)	PD vs. PDmin	PD vs. PDmax	PDmin vs. PDmax
Perm	−0.29	0.21	0.5	0.0033	0.0542	0.0253
RS	−0.27	0.09	0.36	0.0151	0.0723	0.0527
Wilcoxon test (*p*) Perm vs. RS	0.893	0.1005				
Delta	−0.59	−0.12	0.71	0.0002	0.1067	0.0023
RS	−0.7	0	0.7	0.0006	0.2061	0.0172
Wilcoxon test (*p*) Delta vs. RS	0.0614	0.0036				
Mann–Whitney test (*p)* Perm vs. Delta	0.0402	0.0023				

Abbreviations: RS—iso-osmotic Ringer’s solution. *p* < 0.05 is considered statistically significant (in red).

## Data Availability

The data will be made available upon request; email: igaholynska@cm.umk.pl.

## Supplementary Materials

The following supporting information can be downloaded at https://www.mdpi.com/article/10.3390/biom14121491/s1: Figure S1: The comparison of the initial transepithelial electric resistance (R, Ω/cm^2^) measured at stationary conditions of the skin specimens, incubated in: iso-osmotic Ringer solution (Control), Permethrin (Perm, 0.01 mM) and Deltamethrin (Delta, 0.01 mM). Figure S2: The comparison of the final transepithelial electric resistance (R, Ω/cm^2^) measured at stationary conditions of the skin specimens, incubated in: iso-osmotic Ringer solution (Control), Permethrin (Perm, 0.01 mM) and Deltamethrin (Delta, 0.01 mM). Figure S3: The comparison of the initial transepithelial electric potential (PD, mV) measured at stationary conditions of the skin specimens, incubated in: iso-osmotic Ringer solution (Control), Permethrin (Perm, 0.01 mM) and Deltamethrin (Delta, 0.01 mM). Figure S4: The comparison of the final transepithelial electric potential (PD, mV) measured at stationary conditions of the skin specimens, incubated in: iso-osmotic Ringer solution (Control), Permethrin (Perm, 0.01 mM) and Deltamethrin (Delta, 0.01 mM).
