# Supplementary material for: A 30-Min Exposure on Permethrin and Deltamethrin Modifies Ion Transport Pathways in the Skin
Source: Biomolecules. 2024 Nov 22;14(12):1491. doi: 10.3390/biom14121491 (PMC11672964; doi:10.3390/biom14121491)
Supplement: Supplementary file 1 [file biomolecules-14-01491-s001.zip › biomolecules-3158489-supplementary.pdf]

Type of the Paper: Article

Title: A 30-min exposure on permethrin and deltamethrin modifies ion transport pathways in the skin.

Karolina Szewczyk-Golec[1], Katarzyna Mądra-Gackowska[2], Łukasz Szeleszczuk[3], Jan Szczegielniak[4,5], Iga Hołyńska-Iwan[6]*

[1] Department of Medical Biology and Biochemistry, Faculty of Medicine, Ludwik Rydygier Collegium Medicum in Bydgoszcz, Nicolaus Copernicus University in Torun, Bydgoszcz, Poland
[2] Department of Geriatrics, Faculty of Health Sciences, Ludwik Rydygier Collegium Medicum in Bydgoszcz, Nicolaus Copernicus University in Torun, Bydgoszcz, Poland
[3] Department of Organic and Physical Chemistry, Faculty of Pharmacy, Medical University of Warsaw, Warsaw, Poland
[4] Physiotherapy Department, Faculty of Physical Education and Physiotherapy, Opole University of Technology, Opole, Poland
[5] Ministry of Internal Affairs and Administration's Specialist Hospital of St. John Paul II, Glucholazy, Poland
[6] Department of Pathobiochemistry and Clinical Chemistry, Faculty of Pharmacy, Ludwik Rydygier Collegium Medicum in Bydgoszcz, Nicolaus Copernicus University in Torun, Bydgoszcz, Poland
* Correspondence: Iga Hołyńska-Iwan, Ph. D., Prof. NCU, email: igaholynska@cm.umk.pl; Laboratory of Electrophysiology of Epithelial Tissue and Skin, Department of Pathobiochemistry and Clinical Chemistry, Collegium Medicum in Bydgoszcz Nicolaus Copernicus University in Torun, address: M. Skłodowskiej-Curie 9, 85 – 094 Bydgoszcz, Poland

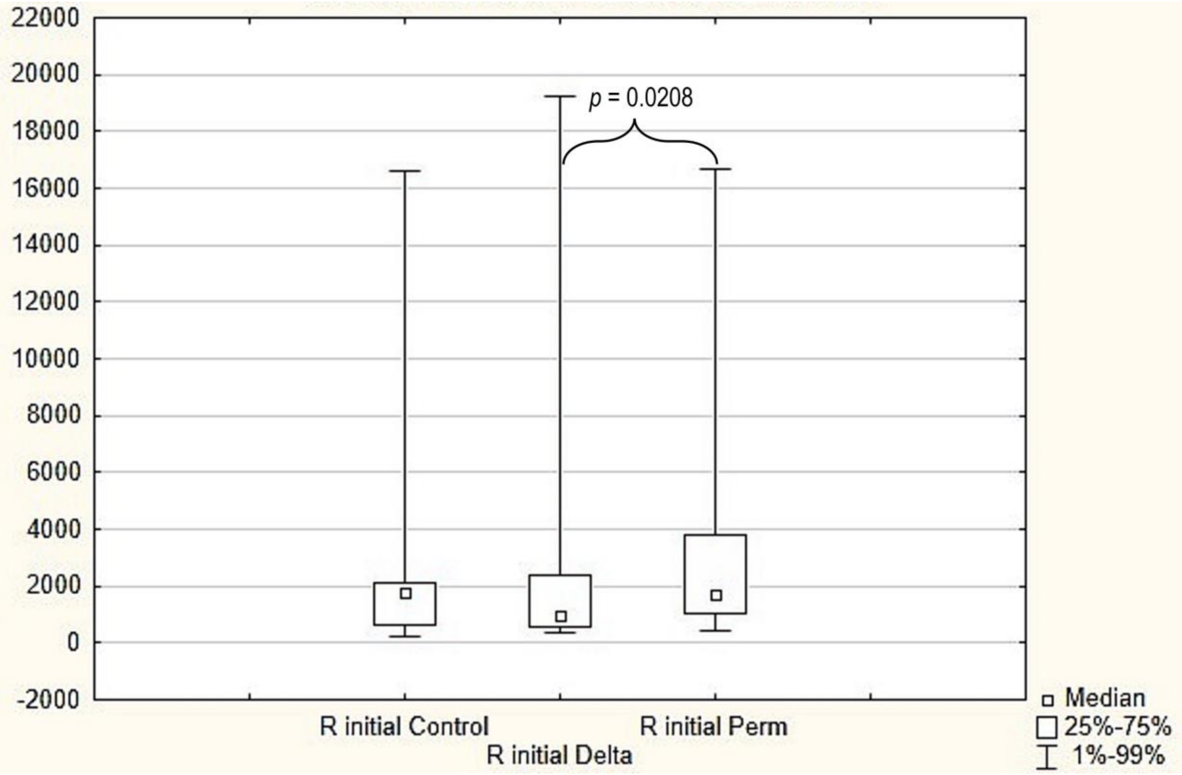

Figure S1 The comparison of the initial transepithelial electric resistance (R, Ω/cm^2) measured at stationary conditions of the skin specimens, incubated in: iso-osmotic Ringer solution (Control), Permethrin (Perm, 0.01 mM) and Deltamethrin (Delta, 0.01 mM).

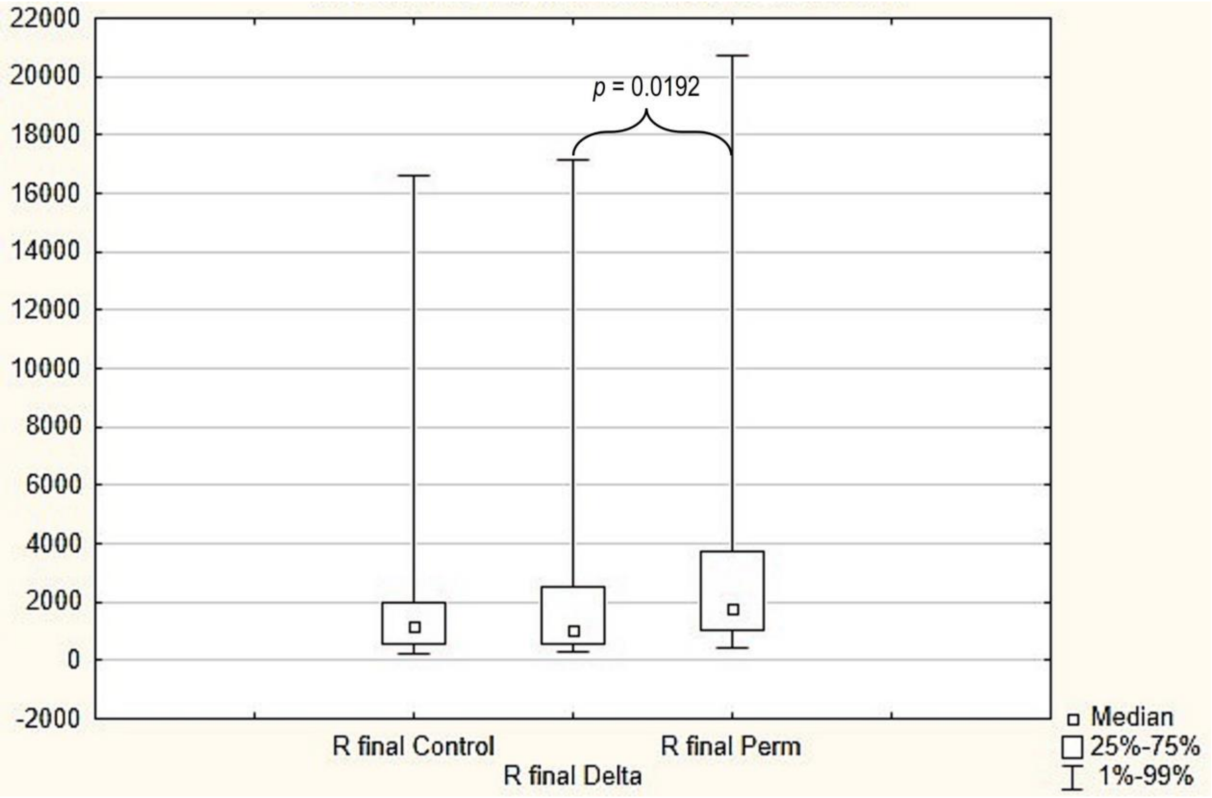

Figure S2 The comparison of the final transepithelial electric resistance (R, Ω/cm^2) measured at stationary conditions of the skin specimens, incubated in: iso-osmotic Ringer solution (Control), Permethrin (Perm, 0.01 mM) and Deltamethrin (Delta, 0.01 mM).

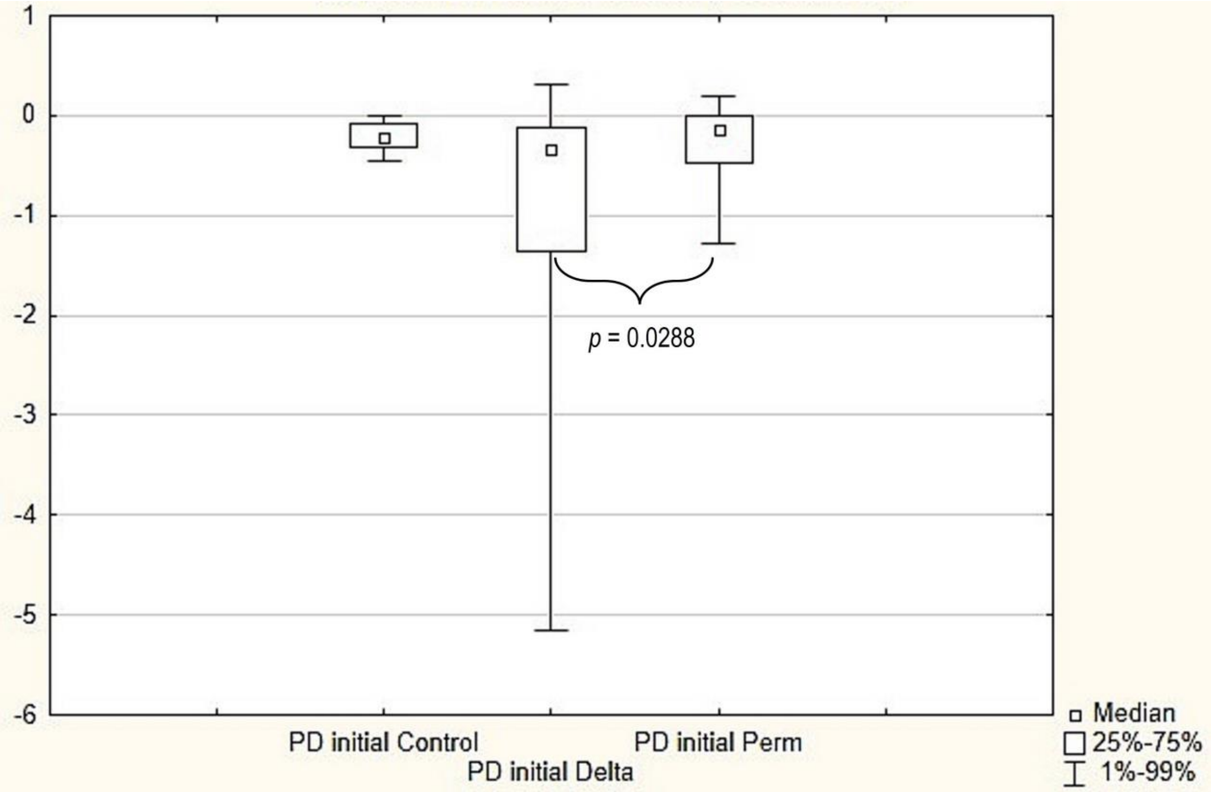

Figure S3 The comparison of the initial transepithelial electric potential (PD, mV) measured at stationary conditions of the skin specimens, incubated in: iso-osmotic Ringer solution (Control), Permethrin (Perm, 0.01 mM) and Deltamethrin (Delta, 0.01 mM).

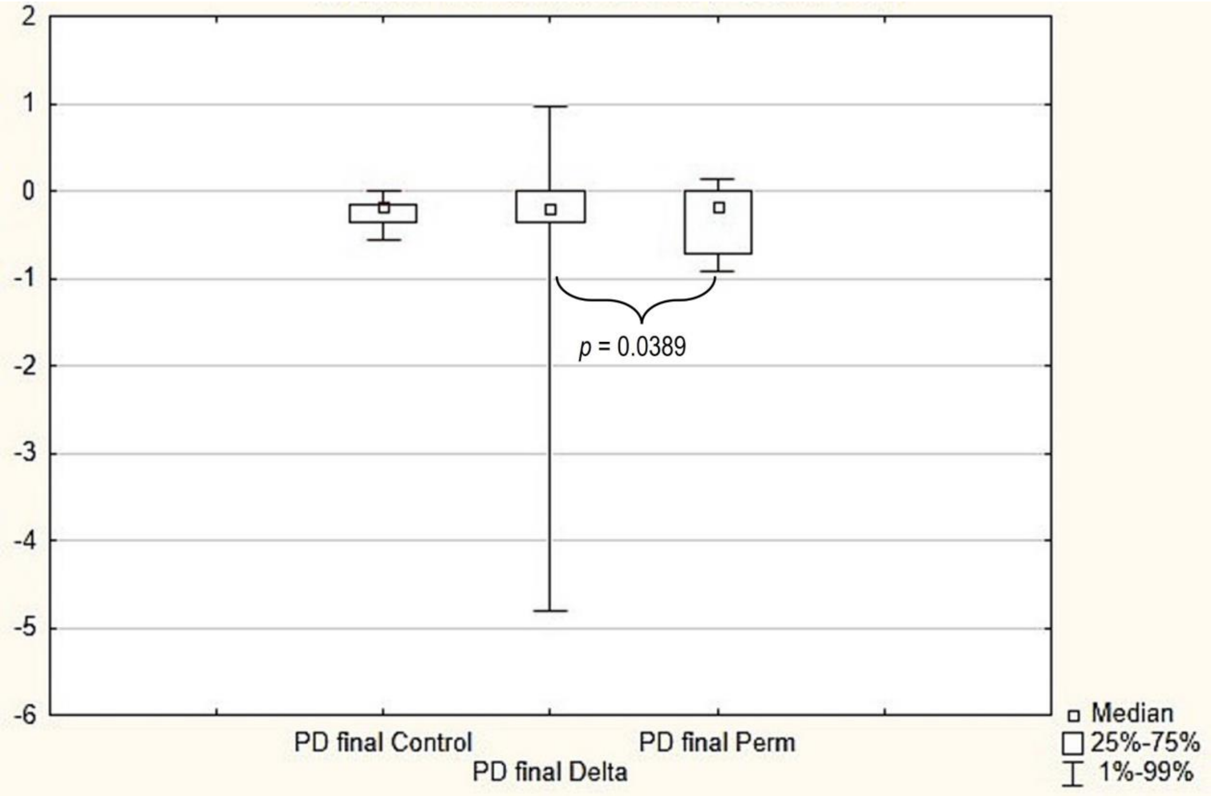

Figure S4 The comparison of the final transepithelial electric potential (PD, mV) measured at stationary conditions of the skin specimens, incubated in: iso-osmotic Ringer solution (Control), Permethrin (Perm, 0.01 mM) and Deltamethrin (Delta, 0.01 mM).
